# Multimodal Imaging Observation in Different Progressive Types of Bietti Crystalline Dystrophy

**DOI:** 10.1155/2022/7426052

**Published:** 2022-05-24

**Authors:** Shengjuan Zhang, Lifei Wang, Yanhui Wang, Yanxia Shang, Xin Wang, Lizhen Ma, Zanzhang Yang, Chen Xing, Xiaoyan Peng

**Affiliations:** ^1^Beijing Institute of Ophthalmology, Beijing Tongren Eye Center, Beijing Tongren Hospital, Capital Medical University, Beijing 100730, China; ^2^Hebei Provincial Eye Hospital, Hebei Provincial Key Laboratory of Ophthalmology, Hebei Provincial Eye Institute, Xingtai, Hebei 054001, China

## Abstract

**Objective:**

The aim of the study is to observe the difference in progression between type 1 and type 2 Bietti crystalline dystrophy (BCD) using multimodal imaging.

**Methods:**

A retrospective clinical study was performed with six BCD patients who underwent multimodal imaging twice in Hebei Provincial Eye Hospital from October 2015 to December 2020. Multimodal imaging includes color fundus photography, fundus autofluorescence (AF), infrared autofluorescence (IRAF), fundus fluorescein angiography (FFA), and spectral domain optical coherence tomography (SD-OCT). The fundus lesion progression difference was observed in 3 patients with type 1 BCD and 3 patients with type 2 BCD.

**Results:**

In type 1 BCD, the range of hypoautofluorescence (hypo-AF), hypoinfrared autofluorescence (hypo-IRAF), and hypofluorescence in the posterior pole was enlarged, and FFA showed that the lesions in the posterior pole and periphery extended to the middle periphery. SD-OCT revealed retinal and choroidal thinning, progressive loss of the outer nuclear layer and ellipsoid zone, and reduction of the choroid macrovascular diameter. In type 2 BCD, the range of hypo-AF was enlarged, but there was no significant change in the macula area. The uniform hypo-IRAF in the posterior pole showed no significant change. FFA showed no significant change with the progression of the disease in the macula area and the hypofluorescence around it expanded. SD-OCT revealed no obvious change in the macula area.

**Conclusions:**

The retinal choroid atrophy in the macula area of type 1 BCD continued to worsen, and the choroid great vessels became narrower. The macular lesions of type 2 BCD can remain unchanged for a long time.

## 1. Introduction

Bietti crystalline dystrophy (BCD) was first reported by Bietti et al. in 1937 [1]. It is characterized by numerous tiny sparkling yellow-white crystalline deposits in the posterior pole retina, retinal pigment epithelium (RPE) atrophy, and choroid sclerosis [2]. Current literature studies mainly focus on genetic diagnosis [3], cross-sectional imaging studies [4, 5], and case reports [6, 7]. Follow-up observation studies are rare, and most of them are case reports [8, 9]. Our previous study found that patients with BCD can be divided into type 1 and type 2 based on progressive changes shown by fundus fluorescein angiography (FFA) [10]. Multimodal imaging has been proven to play a very positive role in the diagnosis of BCD and the differentiation of BCD from other degenerative retinal and choroidal diseases [4]. In this research study, multimodal imaging was used to observe the different characteristics of lesion progression in patients with type 1 and type 2 BCD.

## 2. Patients and Methods

### 2.1. Patients

The implementation of all research methods in this study followed the provisions of the Helsinki Declaration, the Ethics Committee of Beijing Tongren Hospital and the Ethics Committee of Hebei Provincial Eye Hospital. This study included 6 BCD subjects who underwent fundus color photography, autofluorescence (AF), infrared autofluorescence (IRAF), FFA, and spectral domain optical coherence tomography (SD-OCT) twice in Hebei Provincial Eye Hospital from October 2015 to December 2020. The clinical features and imaging changes of these patients were retrospectively analyzed.

Written informed consent was obtained from each patient before peripheral venous blood was drawn for genomic DNA extraction and mutation screening of the CYP4V2 gene by direct sequencing. The 6 subjects were unrelated Chinese patients, and all diagnosed with BCD by genetic testing.

For convenience of description, the 6 patients are referred to as P1, P2, P3, P4, P5, and P6. The interval between the two multimodal imaging was 1 year for P1 and P2, 3 years for P3, and 2 years for P4–P6.

Our previous study divided BCD into two types based on the observation of FFA progression in 12 patients with BCD [10]. In type 1, patchy RPE atrophy occurred first in the macula area, followed by patchy choroidal capillaries in the macula area, and then RPE atrophy occurred in the peripheral retina. Both macula area and peripheral lesions spread to the middle periphery, and finally, whole retina and choroid were atrophied. In type 2, RPE atrophy occurred in the posterior pole and peripheral fundus, followed by atrophy of the choroidal capillaries around the macula area. Both macula and peripheral lesions spread to the middle periphery; finally, whole retina and choroid were atrophied. However, RPE lesions of the macula area can remain for a long time until the advanced stage of BCD without choroidal capillary atrophy. According to the results, P1, P2, and P3 were type 1, and P4, P5, and P6 were type 2.

## 3. Methods

All patients underwent a complete ophthalmic examination, including the best corrected visual acuity (BCVA), slit lamp microscopy, Goldman flattening tonometry dilated fundus examination, and fundus photography (Kowa, Nonmyd 7, Kowa, Japan). AF, IRAF, and FFA images were obtained with a confocal scanning laser ophthalmoscope (Heidelberg Spectralis, Heidelberg Engineering, Heidelberg, Germany) with a 55 × 55° field lens.

**Figure 1:**
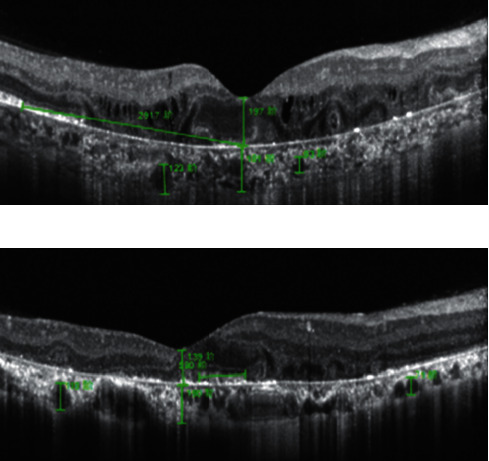
OCT parameter measurement in type 1 patients. (a) Measurement methods of the right eye parameters of P1. (b) Measurement method of parameters of both eyes of P2 and P3, and the left eye of P1.

SD-OCT images were obtained by spectral-domain optical coherence tomography (Heidelberg Engineering, Heidelberg, Germany). Central macular thickness (CMT) and subfoveal choroid thickness (SCT) were measured in the B-scan flat through the macula fovea. Two large choroid vessels that easily measured were selected for measurement of the vessel diameter, one at the nasal side and one at the temporal side of the choroid below the fovea. The difference of vessels' diameter between the first and last examination was calculated, and the change of vessels' diameter was calculated. The ellipsoid zone (EZ) of the fovea in the right eye of P1 was missing, but the remaining EZ was visible in the temporal side of the fovea. Therefore, the distance between the Bruch membrane of the fovea and the residual EZ of the temporal side was measured, and the difference between the first and last measured values was calculated (Figure 1(a)). A small amount of EZ was found in the fovea of the left eye of P1 and both eyes of P2 and P3. The width of the EZ was measured at the first and last visit, and the difference between them was calculated (Figure 1(b)). In type 2 BCD, the EZ in the macula area is missing. All values were measured by two physicians.

## 4. Results

### 4.1. Basic Patient Characteristics, Genetic Diagnoses, and Color Fundus Photography Results

The age of six patients ranged from 30 to 46 years at initial diagnosis. The BCVA of the type 1 (2 males and 1 female) and type 2 (2 males and 1 female) BCD patients decreased significantly. The genetic data of the patients are shown in Table 1.

**Figure 2:**
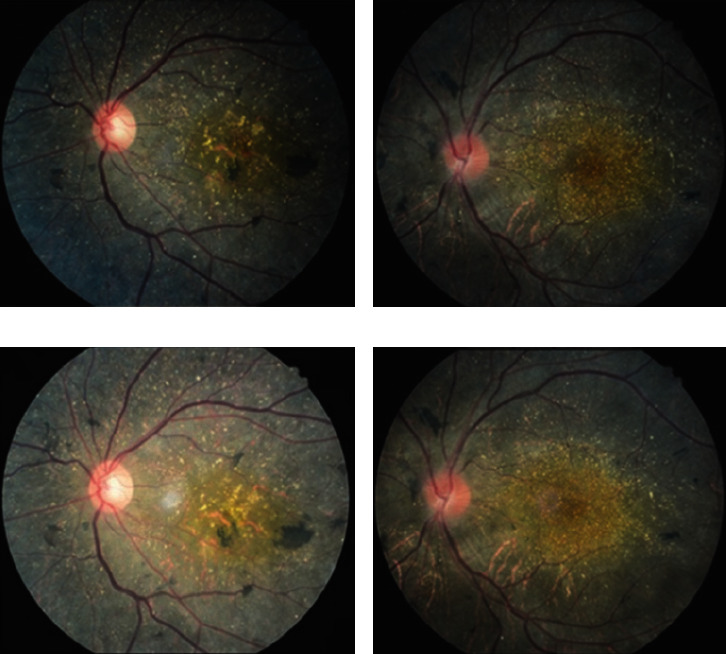
Comparison of first and last fundus color photographs in patients with type 1 and type 2 BCD. (a, c): Color fundus images of the left eye of type 1 patient P2 at the first and last visit, respectively, show a significant reduction of crystalline material. (b, and d): Retinal changes were not obvious in the macula area of the left eye of type 2 patient P4. The crystalline deposits of the retina surrounding macula area were significantly reduced.

With the progression of the disease, the crystalline deposits in the posterior pole fundus of patients with type 1 BCD decreased significantly (Figures 2(a) and 2(c)). In type 2 patients, there was no significant change of crystalline deposits in the macula area, and the number of crystalline deposits surrounding the macula area decreased significantly (Figures 2(b) and 2(d)).

### 4.2. AF and IRAF Results

**Figure 3:**
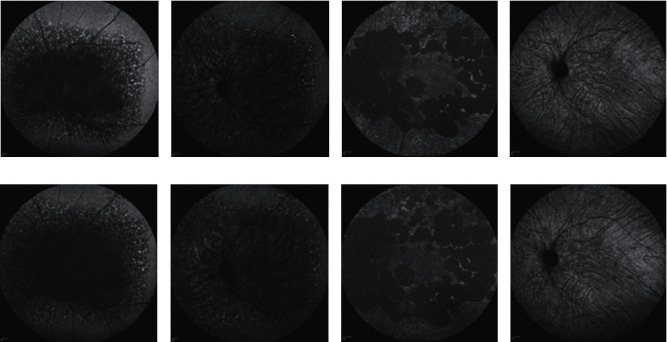
Comparison of first and last fundus autofluorescence (AF) and infrared autofluorescence (IRAF) images in patients with type 1 and type 2. (a, e): First and last AF images, respectively, of the left eye of type 1 patient P1, showing enlarged area of hypo-AF at the posterior pole. (b, f): First and last IRAF images, respectively, of the left eye of P1, showing the expansion of hypo-IRAF area in the posterior pole. (c, g) First and last AF images, respectively, of the left eye of type 2 patient P4, showing that the range of hypo-AF around the macula area expanded at the posterior pole, with no significant AF change in the macula area. (d, h): First and last IRAF images, respectively, of the left eye of P4, showing no significant change of the posterior pole.

The AF and IRAF of both eyes of the BCD patients were basically the same. The range of hypo-AF in P1–P6 expanded. The hypo-AF region is formed by the combination of multiple small patches of hypo-AF (Figures 3(a), 3(c), 3(e), and 3(g)). In type 1 patients, hypo-AF was observed in the macula area of the retina, and spot-like hyper-AF was observed around the hypo-AF (Figures 3(a) and 3(e)). Different from type 1 patients, RPE-choroidal capillary atrophy in the macula area was light in type 2 patients. The first and last AF in the macula area did not change significantly (Figures 3(c) and 3(g)).

The IRAF images of type 1 patients showed hyper-IRAF spots at the edge of the hypo-IRAF, and there were fewer hyper-IRAF spots than hyper-AF spots (Figures 3(b)and 3(f)). The posterior pole of patients with type 1 lesions beyond the 55° range and the posterior pole of all the patients with type 2 lesions were hypo-IRAF, which did not change significantly with disease progression (Figures 3(d) and 3(h)).

### 4.3. FFA Results

**Figure 4:**
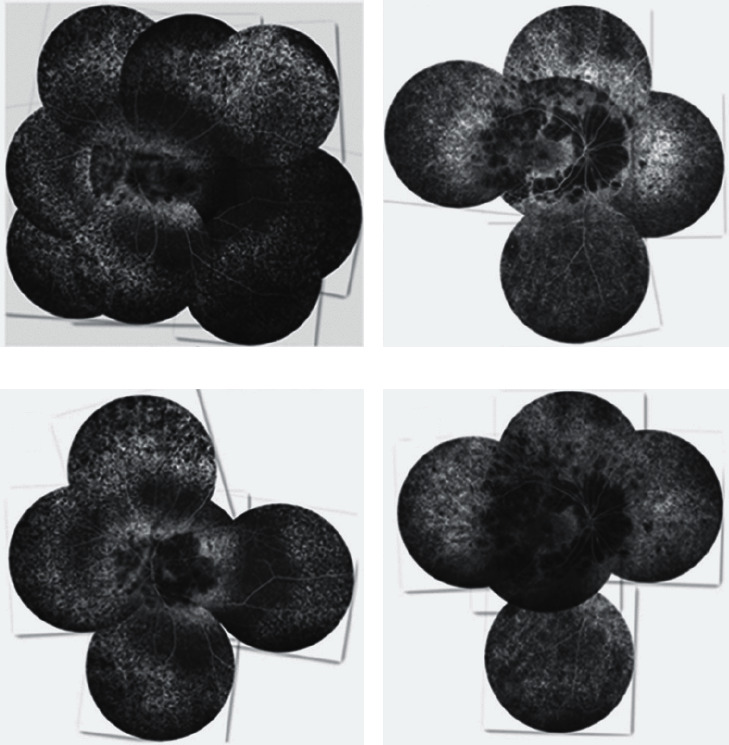
Comparison of first and last fundus angiography in patients with type 1 and type 2 BCD. (a) and (c): Puzzles of the first and last angiography images, respectively, of left eye of type 1 patient P1, showing that the hypofluorescence of the posterior pole expanded, and the peripheral and posterior pole lesions developed to the mid-peripheral. (b) and (c): Puzzles of the first and last fundus angiography images, respectively, of the right eye of type 2 patient P4, showing no significant changes in the macula area and the hypofluorescence around macula area enlarged.

The regularity of FFA progression in BCD patients has been reported in detail in our previously published paper [10]. The specific classification method has been described in the Patients and Methods section of this paper. In this paper, P1, P2, and P3 belong to type 1, while P4, P5, and P6 belong to type 2, and the FFA progression changes are the same as in our previous study [10] (Figure 4).

### 4.4. OCT Results

**Figure 5:**
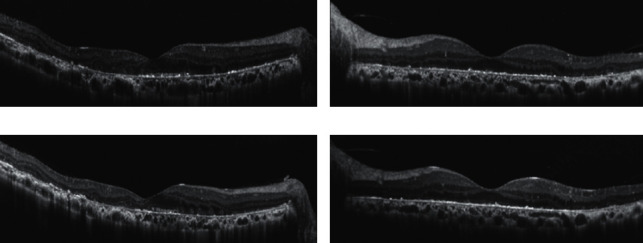
Comparison of first and last SD-OCT images from patients with type 1 and type 2 BCD. (a) and (c): First and last SD-OCT images, respectively, of the right eye of type 1 patient P2, showing thinning of retina and choroid in macula area and shortening of EZ in fovea. (b) and (d): First and last SD-OCT images, respectively, of the left eye of type 2 patient P4, showing no significant changes in macula area.

Type 1 patients had varying degrees of atrophy in the outer nuclear layer, EZ, interdigitation zone, and RPE, with partial loss of reflected light. The choroid thinned. The indicators included CMT, SCT, residual EZ, and reduction of the choroid nasal and temporal vessel diameter (except that the left eye of P1 was thickened with CMT due to increased retinal edema). There was no EZ residue in the fovea of the right eye of P1, so the distance between the remnant of the EZ at the temporal side of the fovea and the Bruch membrane at the fovea was measured. The last distance was 165 *μ*m greater than the first, indicating that EZ was also lost progressively in the right eye of P1. In type 2 patients, the EZ, the interdigitation zone, and part of the RPE were absent in the macular area, and the external limiting membrane was clearly visible (Figure 5). Except for the exclusion of the left eye of P5 due to complicated choroidal neovascularization and the 17 *μ*m thinning of CMT in the right eye of P4, the changes in other measured values were all small. Compared with the thickness of the retina and choroid, the changes were miniscule. Therefore, we believed that the macular area of type 2 patients had basically no changes or no obvious changes (Table 2).

## 5. Discussion

The 6 patients observed in this study ranged in age from 30 to 46 years, which is the common age of BCD onset. The results of gene mutation of the 6 patients were all BCD-related gene mutation sites [11, 12].

Color fundus photographs of type 1 BCD showed a decrease in crystalline deposits with the progression of disease, consistent with previous reports [9]. The yellow-white crystalline deposits are caused by the abnormal expression of the CYP4V2 gene in the human retina and RPE [13], which decreases with the deterioration of the retina and RPE atrophy. The crystalline substances in the macula area of type 2 BCD did not change significantly with the progression of disease, and the crystalline substances surrounding the macula area decreased, suggesting that the changes of RPE in the macula area were not obvious. To our knowledge, this phenomenon has not been reported in the previous literature.

It is known that AF mainly reflects the fluorescence of lipofuscin [14]. The locations of hypo-AF in type 1 and type 2 patients are different. Hypo-AF of type 1 extends from the macula to the periphery. In the type 2 macula area, the AF changes were not obvious. The patchy hypo-AF around the macula area, corresponding to the position of the vascular arch and the nasal side of the optic disc, became bigger and expanded outwards. AF is not affected by fluorescent staining, transparent fluorescence, or fluorescence leakage caused by sodium fluorescein, it reflects the most real spontaneous fluorescence of fundus tissues and can clearly reflect the lipofuscin condition of the fundus tissues. From the change of lipofuscin, it can also reflect the atrophy of the RPE-choroidal capillaries. Therefore, it can be seen that the retina and the choroid of the macula area in type 2 patients have no significant change. We also found that the hypo-AF area in the posterior pole of type 1 and type 2 patients did not develop from one place; rather, it was multiple patchy hypo-AF regions that had expanded and become connected. This was consistent with the findings of Kojima et al. [15], who found that the RPE between the patchy hypo-AF regions was relatively less damaged and still retained some functions. IRAF mainly reflects the fluorescence of melanin [16]. The macula area and the area surrounding it were hypo-IRAF in type 2 BCD, indicating that melanin damage in the pigment epithelial cells and the choroid is the same in the macula area and other areas of the posterior pole.

Retinal and choroidal atrophy in the last FFA examination of 6 BCD patients was significantly worse than that in the first FFA examination, which was consistent with previous reports [8, 9]. Both eyes had similar FFA imaging in all 6 patients. Previous imaging studies of BCD suggested that BCD lesions developed from the posterior pole to the periphery in an eccentric manner [17, 18]. However, what we found was different. We found that BCD lesions first appeared in the posterior pole and then in the peripheral retina. Next, the posterior pole and peripheral lesions spread to the middle periphery, and eventually the whole fundus was involved. The difference between type 2 and type 1 is that the atrophy of choroidal capillaries in type 2 BCD starts not in but around the macula area, and the retinal and choroidal atrophy of the macula area is mild and can remain for a longer time.

In this study, the changes in the retina and choroid in BCD patients were quantified by SD-OCT. In type 1 patients, both the retina and choroid thinned significantly, and the diameters of choroidal great vessels narrowed. Studies have reported that the blood flow density of choroidal capillaries in BCD patients decreases as measured by optical coherence tomography angiography [19]. According to our study, the great choroid vessels also atrophy. In type 2 patients, there was little change in the retina and choroid in the macula area. The SD-OCT was consistent with the results of fundus color photograph, AF, IRAF, and FFA. That is, the lesions in the macula area of type 2 BCD can remain in a milder RPE-choroidal capillary atrophic state for a long time. Through the comparison of gene mutation sites, it was found that P1 of type1 BCD and P5 of type 2 BCD had the same mutation sites, but the phenotypes of the two patients were different, so there was no direct correlation between the genotype and phenotype. Further studies are needed to explain the differences between type 1 and type 2 phenotypes.

The limitation of this study was that the number of eyes included was small and the observation time was not very long. However, if the rarity of the disease and previous literature are considered, this was the first study to observe the progress of BCD by multimodal imaging, and the number of cases reported was relatively large.

## 6. Conclusions

In conclusion, in addition to atrophy of the retina and choroid capillary layers, the diameter of the choroid great vessels in type 1 BCD was also significantly reduced. In type 2 BCD patients, retinopathy was significantly aggravated, except for the macula area. Noninvasive examination of AF, IRAF, and SD-OCT have great advantages in assessing BCD progression and changes.

## Figures and Tables

**Table 1:** Genetic and consanguinity status.

Patient	Genetic analysis		Consanguinity
	Allele 1	Allele 2	
P1	c.802-8_810del17insGC	c.802-8_810del17insGC	N
P2	c.992A > C, p.H331P	c.992A > C, p.H331P	N
P3	c.802-8_810del17insGC	g.2979A > G; chr4:187115652A > G	N
P4	c.802-8_810del17insGC	c.332T > C; p.l111T	N
P5	c.802-8_810del17insGC	c.802-8_810del17insGC	N
P6	c.802-8_810del17insGC	c.401G > A, p.G134E	N

N, no.

**Table 2:** Comparison of first and last SD-OCT results (Counting unit: *μ*m).

Patients	CMT		SCT		Vascular diameter (temporal)		Vascular diameter (nasal)		Ellipsoid zone (fovea)		Ellipsoid zone (fovea to the temporal ellipsoid zone)	
	OD	OS	OD	OS	OD	OS	OD	OS	OD	OS	OD	OS
P1	51	−12	72	88	28	32	38	27	—	169	−165	—
P2	15	73	29	25	20	40	19	17	476	393	—	—
P3	38	6	12	28	16	9	31	14	480	561	—	—
P4	17	0	4	9	8	4	0	0	N	N	—	—
P5	3	—	6	—	0	—	0	—	N	N	—	—
P6	4	2	8	2	2	4	0	2	N	N	—	—

All data in the table are the difference between the first visit and the last visit measurements. CMT: central macular thickness; SCT: subfoveal choroidal thickness; vascular diameter (temporal): the diameter of a large choroid vessel in the temporal of subfoveal choroid; vascular diameter (nasal): the diameter of a large choroid vessel in the nasal of subfoveal choroid; OD, right eye; OS, left eye; F: first visit; L: last visit; N: no.

## Data Availability

The data used to support the findings of this study are available from the corresponding author on request.
